# Identifying Unique Contributions of the Coach–Athlete Working Alliance, Psychological Resilience and Perceived Stress on Athlete Burnout among Norwegian Junior Athletes

**DOI:** 10.3390/sports7090212

**Published:** 2019-09-13

**Authors:** Emilie F. W. Raanes, Maria Hrozanova, Frode Moen

**Affiliations:** 1 Centre for Elite Sports Research, Kavli Institute for Systems Neuroscience, Faculty of Medicine and Health Sciences, Norwegian University of Science and Technology, 7491 Trondheim, Norway; 2 Centre for Elite Sports Research, Department of Neuromedicine and Movement Science, Faculty of Medicine and Health Sciences, Norwegian University of Science and Technology, 7491 Trondheim, Norway; 3 Centre for Elite Sports Research, Department of Education and Lifelong Learning, Faculty of Social and Educational Sciences, Norwegian University of Science and Technology, 7491 Trondheim, Norway

**Keywords:** athlete burnout, coach-athlete alliance, psychological resilience, perceived stress

## Abstract

The main purpose of the current study was to examine how the coach-athlete working alliance, psychological resilience and perceived stress are uniquely associated with burnout among junior athletes in sport. A sample of 670 Norwegian junior athletes practicing a variety of sports participated in the study. A hierarchical multiple regression analysis showed that the bond dimension of the working alliance, the protective factors ‘planned future’ and ‘structured style’, as well as perceived stress, all contributed uniquely to the explanation of athlete burnout. A dominance analysis identified perceived stress to have the strongest relative influence on athlete burnout among the set of variables investigated in this study. The findings are discussed in terms of applied implications and possible future research.

## 1. Introduction

High schools specialized for elite sports are aimed at promoting both personal growth and enhancements in sport-specific capabilities [1]. However, intense physical and psychological loads over time may lead young athletes to experience such loads as chronic stress [2,3,4]. High training loads, uncertainty associated with selection processes, biological changes occurring during puberty, possible role conflicts, and pressure to perform well both in sport and at school are potential stressors [5]. The athletes may also become ill or injured and thereby experience performance impairments. Furthermore, winning an important competition could also be a stressor in the sense that it requires the individual to positively adapt to the heightened expectations related to success [6]. Although stress is an inevitable part of competitive sports, it is well-documented that chronic stress is detrimental to athletes’ personal growth and sport-specific performance and may lead to the non-functional state known as athlete burnout [7,8,9,10]. Research shows that up to 9% of competitive adolescent athletes might be afflicted with symptoms of burnout [11].

Importantly, sport coaches have a responsibility to prepare their athletes both physically and mentally for athletic training and competitions [12], and an effective coach-athlete working alliance is considered crucial in order to cultivate an athlete’s personal growth in sport [13,14,15,16,17]. Coaches may, therefore, have a significant impact on athletes’ ability to overcome adversity and adapt during times of stress—a mental capacity defined as psychological resilience [18]. The main purpose of the current study is, therefore, to examine how the coach–athlete working alliance, psychological resilience and perceived stress are uniquely associated with burnout among junior athletes in sport.

Smith’s (1986) cognitive-affective model claims that stress appraisals occur when perceived situational demands exceed the athlete’s personal and socio-environmental resources available to cope, leading to physiological and psychological responses and, finally, behavioural and coping responses [2]. In this theoretical framework, athlete burnout is considered to be the product of chronic stress. In Raedeke’s (1997) conceptual definition, athlete burnout is a multifaceted syndrome comprising three dimensions: (1) Emotional and physical exhaustion, characterized by feelings of fatigue from training, competitions and different psychological hassles, (2) reduced sense of accomplishment, identified as the feeling of being unable to achieve personal goals and performing below expectations, and (3) sport devaluation, referring to a loss of interest and care for one’s performance and the sport environment [19,20].

The progress of athletic performance is brought about by changes in skills and capacities, and the coach-athlete relationship may, therefore, be considered to be a change-inducing relationship [21]. Based on the work of Bordin (1979), such a relationship is defined as a working alliance between the helper and the help-seeker [22]. The working alliance consists of three interdependent components: Bond embraces the positive personal attachments between the helper and the help-seeker including issues such as mutual trust, acceptance and confidence [22,23,24]; Goal refers to the helper and the help-seeker mutually agreeing on the aims that are the target of the helping relationship; Task refers to the activities that the partnership will engage in to facilitate change, and both parties should perceive these tasks as applicable and beneficial [22,23,24]. Measuring these characteristics, a coach–athlete Working Alliance Inventory (WAI) has been introduced in the sport setting [21,25].

The common premise behind the term ‘psychological resilience’ is positive adaptation in the face of difficulties, adversity or long-term stress [18]. Fletcher and Sarkar (2012) offered the first definition of the resilience in sport as “the role of mental processes and behaviour in promoting personal assets and protecting an individual from the potential negative effect of stressors” [26]. As protective psychological characteristics may develop and the availability of resources is likely to change over time, sport psychologists have argued that coaches and practitioners will have the greatest promotive impact on athletes’ resilience by (a) taking maximum advantage of their existing protective factors, (b) identifying deficits in protective factors, and (c) fostering the development of resilient qualities and characteristics [26,27]. In this context, the term ‘protective factor’ is used as a generic term for intrapersonal and (perceived) interpersonal resources that enhance positive adaptation in individuals exposed to prolonged stress or adversity. A variety of intra- and interpersonal factors may help athletes adapt to the stressors and setbacks encountered in sports [26,28], and protective factors have further been suggested to operate by influencing athletes’ challenge appraisal and meta-cognitions [26]. Accordingly, the psychological response to stress is subjective and likely to be determined by cognitive appraisal processes [29]. 

The ultimate goal of the coach-athlete relationship is to help athletes become competitive in their sports. On this evolving journey, stress and setbacks are inevitable. Having the ability to withstand stress and view stress as an opportunity for self-growth, is, therefore, necessary for athletes to enter positive developmental trajectories and become mentally prepared for competitions. Consequently, the purpose of the coach role is not only to assist athletes in developing physical skills but also to help them develop psychological resilience [30]. Research suggests that this kind of personal growth may occur only after athletes have experienced significant stress or adversity and importantly, gone through a process of coping [31,32]. An ineffective coach-athlete working alliance may have a negative effect on the athlete’s cognitive appraisals, and perceived situational demands are then more likely to exceed the athlete’s resources available to cope. The coach–athlete relationship may even become an additional source of stress itself and thereof, contribute to distress [33]. However, a high-quality working alliance may promote the development of both resilient qualities and sport-specific skills. Ultimately, athletes are more likely to make positive appraisals of stressors if their coach provides them with adequate social resources, and the social support provided by the coach may such constitute an important resource for coping with stress. Effective coping may contribute to build psychological resilience, which in turn represents the overall capacity of athletes to protect themselves from chronic stress and stress-related outcomes such as athlete burnout. The specific characteristics of the individual such as sex and level of athletic ambition may also affect these processes and contribute to determine an athlete’s susceptibility to burnout. First, previous studies have reported sex differences in protective factors and stress levels [34,35,36]. Second, a desire to pursue long-term goals (i.e., high ambitions) may be necessary for young athletes to persist in spite of difficulties or negative outcomes, build psychological resilience and eventually reach their full athletic potential. Further, a high-quality coach–athlete working alliance may be important for athletes to cope and remain ambitious. On this basis, there is reason to believe that both sex and level of athletic ambition may have effects on the variables investigated in this study.

Estimates suggest that 30% of Norwegian youth aged between 16 and 19 years old participate in organized sport [37]. On this basis, organized sport programs are evidently part of the Norwegian culture and play a significant role in youth development. Given the proportion, and possibly growing number, of junior athletes reporting high levels of burnout symptoms [11], it is important to further investigate how burnout is related to factors that may protect individuals from such maladaptive outcomes. To the best of the authors’ knowledge, this is the first study to assess the unique contributions of the coach–athlete working alliance, psychological resilience and perceived stress to individual differences in burnout among junior athletes in sport. Based on the theoretical presentation, the two following hypotheses were developed:

**Hypothesis** **1** **(H1).** *Sex differences as well as group differences based on level of athletic ambition (ambitions to reach elite level in sports, or no such ambitions) exist in the coach–athlete working alliance, psychological resilience, perceived stress and athlete burnout.*

**Hypothesis** **2** **(H2).** *A strong coach–athlete working alliance and higher levels of protective factors of resilience are associated with lower levels of burnout symptoms in young athletes, and higher levels of perceived stress are associated with higher levels of burnout symptoms in young athletes.*

The study reported in this paper is based on a Master’s thesis [38].

## 2. Materials and Methods

A nationwide cross-sectional online survey was conducted over a four-week period. Participants were recruited from Norwegian high schools specialized for elite sports, at which training is included in the academic schedule every day. In total, 1917 junior athletes were invited by email to voluntarily participate in the study. The questionnaire achieved a 35% response rate. Prior to data collection, the study was considered by the Norwegian Centre for Research Data. The data set was collected for the purpose of doing multiple analyses. The study participants were junior athletes (N = 670) attending elite sport high schools (N = 27) in Norway. The sample consisted of 330 males (49.3%) and 340 females (50.7%) whose ages ranged from 16 to 20 years (M = 17.98, SD = 0.89). The participants practiced a variety of sports (N > 18) including both team and individual sports although nearly half of the sample practiced either soccer (18.4%), handball (17.5%) or cross-country skiing (11.3%). Among the junior athletes in this sample, 38.4% performed at a high level while 61.6% performed at a medium or low level at national competitions. The majority of participants (78.2%) had ambitions to become future elite athletes competing at the international level.

The questionnaire measured psychological variables such as the coach–athlete working alliance as perceived by the athletes, protective factors of resilience, perceived stress and athlete burnout, and covered demographic variables such as sex, age, level of ambition (ambitions to become a future elite athlete competing at the international level, or no such ambitions), type of school (public or private) and type of sport.

The Working Alliance Inventory—Short form (WAI-S). A 12-item short form of the WAI was used to assess the quality of the coach–athlete relationship [39]. The WAI-S is composed of both positively and negatively phrased items which are rated on a Likert scale ranging from 1 (“Never”) to 7 (“Always”), and higher scores on the WAI-S reflect more positive ratings of the working alliance. The WAI-S has been shown to possess good construct validity [39]. A Norwegian version of the WAI-S, translated and adjusted for the sport context by Moen and Myhre (2017) [21], was used. Examples of items covering the three dimensions of the working alliance are (1) bond—“There is mutual trust between my coach and me,” (2) goal—“My coach and I work on mutually agreed-upon goals,” and (3) task—“My coach and me agree about the steps I need to take to improve in my sport.” The Cronbach’s alpha coefficients of the three subscales were 0.73, 0.92 and 0.90, respectively.

The Resilience Scale for Adults (RSA). The RSA was used to measure the athletes’ psychological resilience [40]. This 33-item scale evaluates six intra- and interpersonal protective factors of resilience. The items are rated on a seven-point semantic differential response scale in which each item has a positive attribute at one end and a negative attribute at the other end of the scale continuum. The subscale ‘perception of self’ measures an individual’s own confidence in his/her abilities and judgement, self-efficacy and realistic expectations (e.g., “My abilities,” rated from 1, “I am uncertain about,” to 7, “I strongly believe in”). ‘Planned future’ measures an individual’s ability to have a positive outlook, plan ahead and formulate clear goals that are obtainable (e.g., “My future goals,” rated from 1, “I am unsure how to accomplish,” to 7, “I know how to accomplish”). ‘Social competence’ measures an individual’s ability to initiate verbal contact, be flexible in social matters, create new friendships and feel at ease in social settings (e.g., “Meeting new people is,” rated from 1, “difficult for me,” to 7, “something I am good at”). ‘Family cohesion’ measures the degree to which an individual experiences common family values, and loyalty and stability within the family (e.g., “In my family we like to,” rated from 1, “do things on our own,” to 7, “do things together”). ‘Social resources’ measures the degree to which an individual experiences close bonds with friends/family members and external support from friends/family members, and an individual’s ability to provide support (e.g., “Those who are good at encouraging me are,” rated from 1, “no one,” to 7, “some close friends/family members”). ‘Structured style’ measures an individual’s ability to formulate plans for goal achievement, follow regular routines and organize his/her time (e.g., “Rules and regular routines,” rated from 1, “are absent in my everyday life,” to 7, “simplify my everyday life”). Higher scores on the RSA reflect higher levels of protective factors of resilience. The RSA has been shown to be reliable and satisfactory operationalized, and the instrument’s construct and predictive validity have been supported [40,41,42]. The Cronbach’s alpha coefficients of the six subscales were 0.79, 0.77, 0.76, 0.79, 0.81 and 0.63, respectively. 

The Perceived Stress Scale (PSS). To measure the athletes’ perceived stress levels, the PSS was employed [43]. This 14-item scale measures conditions that are central to the stress experience, such as the degree to which respondents find their lives unpredictable, incontrollable and overloading. The PSS examines an individual’s feelings and thoughts during the last month, and for each item, the respondent is asked to indicate how often he/she felt or thought a certain way. The items are both positively and negatively phrased with response options falling on a five-point Likert scale ranging from 0 (“Never”) to 4 (“Very often”), and they are relatively free of content specific to any subpopulation group. Higher scores on the PSS indicate higher levels of perceived stress. The PSS has been shown to possess acceptable psychometric properties [44]. Examples of items are “During the past month, how often have you felt that you were unable to control the important things in your life?” and “In the last month, how often have you been upset because of something that happened unexpectedly?” The Cronbach’s alpha coefficient of the measurement was 0.84 in the current study. 

The Athlete Burnout Questionnaire (ABQ). The 15-item ABQ was used to assess the athletes’ levels of burnout [20,45]. This scale is based on Raedeke’s definition of athlete burnout and accordingly, measures the three key dimensions of the burnout experience. The stem for each item is “How often do you feel this way?” and respondents are asked to rate the extent to which each item addresses their participation motives in sport on a Likert scale ranging from 1 (“Almost never”) to 5 (“Almost always”). The items are both positively and negatively phrased, and higher scores on the ABQ indicate higher levels of burnout. Examples of items covering the three dimensions are (1) emotional and physical exhaustion—“I feel so tired from my training that I have trouble finding energy to do other things,” (2) reduced sense of accomplishment—“It seems that no matter what I do, I don’t perform as well as I should,” and (3) sport devaluation—“I have negative feelings towards sports.” The Cronbach’s alpha coefficients of the three subscales were 0.85, 0.76 and 0.86, respectively. A global burnout score is achieved by averaging the three subscale scores. The Cronbach’s alpha coefficient of the global burnout score was 0.90.

### Data Analysis

Descriptive statistics, including the mean and standard deviation, were calculated for each of the investigated variables. A scale reliability test, Cronbach’s alpha, was run for each subscale. The relevant assumptions were tested prior to running the correlation analyses. The variables were not normally distributed, as assessed by Shapiro–Wilk’s test (*p* < 0.05). Consequently, the associations between the variables were explored by running the non-parametric Spearman’s correlation (r_s_). Subsequently, a series of independent-samples t-tests were conducted to probe for group differences between athletes with ambitions to become future elite athletes and athletes with no such ambitions, as well as between sexes. Based on the results of these t-tests, level of ambition and sex were included in further analysis. 

**Figure 1:**
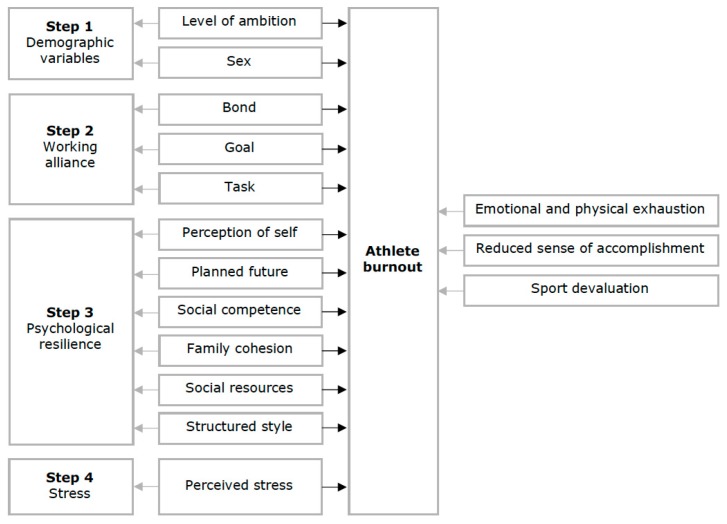
A general conceptual model showing how level of ambition, sex, the three dimensions of the coach–athlete working alliance, the six protective factors of resilience and perceived stress, uniquely and collectively may contribute to explain the variance in athlete burnout.

The assumptions underlying multiple regression were tested. Some outliers were detected by examining the Studentized deleted residuals (> ± 3 SD). However, no leverage or Cook’s Distance values were of concern, hence no outlier was removed from the data. The remaining assumptions were met. To examine how level of ambition, sex, the coach–athlete working alliance, psychological resilience and perceived stress uniquely contribute to the explanation of athlete burnout, a four-step hierarchical multiple regression was run (Figure 1). The control variables, level of ambition and sex, were entered in step 1. The three subscales of the WAI were entered in step 2, followed by the six subscales of the RSA in step 3 and perceived stress in step 4. This order of entry was chosen to reflect causal priority; the effectiveness of the coach–athlete working alliance is likely to influence the athlete’s psychological resilience, which further is likely to be a major determinant of the athlete’s level of stress. 

Lastly, a dominance analysis was conducted to determine the relative importance of the investigated variables in explaining athlete burnout. This statistical method is based on examining the R^2^ values for all possible subset models [46]. The dominance analysis was performed with the DOMIN add-on module [47] in Stata/MP (v. 15, Boston, MA, United States). All other statistical analyses were performed with IBM SPSS (v. 25, Armonk, NY, United States).

## 3. Results

Spearman’s correlations, minimum and maximum scores, means, standard deviations, reliability estimates (α) and the number of survey items that covers each variable, are presented in Table 1. Means and standard deviations of the measurements revealed a moderate to high quality of the coach–athlete working alliance, a moderate to high level of protective factors of resilience, a moderate level of life stress and a moderate level of burnout among the study participants. All measurements had good Cronbach’s alpha coefficients in comparison to Nunnally’s guideline of 0.70 [48], except for the RSA factor ‘structured style’ whose alpha value was somewhat lower (α = 0.63). Some weakness in the subscale ‘structured style’ has also been found in previous studies [42,49]. However, ‘structured style’ has been shown to have high test re-test reliability [42], and scale reliability was, therefore, considered sufficient. Spearman’s correlations indicated (a) moderate negative associations between the three dimensions of the working alliance and athlete burnout, (b) weak to moderate negative associations between the six protective factors of resilience and athlete burnout, and (c) a strong positive association between perceived stress and athlete burnout. All correlations were significant at *p* < 0.01.

Many group differences were found between athletes with ambitions to become future elite athletes and athletes with no such ambitions (Table 2), and between male and female athletes (Table 3).

The full model (Model 4) was statistically significant, R^2^ = 0.47, F(12, 647) = 47.56, *p* < 0.0005, adj. R^2^ = 0.46 and accounted for 47% of the explained variance in athlete burnout with adjusted R^2^ = 46%. A summary of each regression model is presented in Table 4.

Model 1, reflecting level of ambition and sex, accounted for 11% of the explained variance in burnout scores, R^2^ = 0.11, F(2, 657) = 41.49, *p* < 0.0005. The addition of the three subscales of the WAI (Model 2) led to an increase in R^2^ of 0.19, F(3, 654) = 57.41, *p* < 0.0005 and the measured variables thus accounted for 30% of the explained variance in athlete burnout. The addition of the six subscales of the RSA (Model 3) led to an increase in R^2^ of 0.13, F(6, 648) = 24.87, *p* < 0.0005 and at this stage, the regression model accounted for 43% of the explained variance in burnout scores. The addition of perceived stress (Model 4) led to an increase in R^2^ of 0.04, F(1, 647) = 48.56, *p* < 0.0005. 

General dominance was established for all independent variables. Perceived stress (rank 1) was identified to have the strongest relative influence on athlete burnout, followed by the protective factors ‘planned future’ (rank 2) and ‘perception of self’ (rank 3), the bond (rank 4) and task (rank 5) dimensions of the working alliance, level of ambition (rank 6), the goal dimension of the working alliance (rank 7), the protective factors ‘structured style’ (rank 8), ‘social resources’ (rank 9), ‘family cohesion’ (rank 10) and ‘social competence’ (rank 11) and, lastly, sex (rank 12). A summary of the dominance analysis results is presented in Table 5.

## 4. Discussion

The purpose of the current study is two-fold: (1) to investigate potential group differences in the coach–athlete working alliance, psychological resilience, perceived stress and athlete burnout based on sex and level of athletic ambition (H1), and (2) to assess the unique contributions of the coach–athlete working alliance, psychological resilience and perceived stress to individual differences in burnout among junior athletes in sport (H2).

Lending support to H1, many group differences were found on the investigated variables based on sex and level of ambition. Providing support for H2, level of ambition, sex, the bond dimension of the working alliance, the protective factors ‘planned future’ and ‘structured style’, as well as perceived stress, all contributed uniquely to the explanation of athlete burnout. Collectively, these variables accounted for 47% of the explained variance in burnout scores. The results also showed that perceived stress had the strongest relative influence on athlete burnout. The current results give reason to discuss how the measured variables each may play a significant role in the prevention and/or development of burnout in junior athletes.

### 4.1. Sex and Ambition

The unique association between sex and athlete burnout indicates that male athletes are more likely to experience burnout symptoms than female athletes. However, no significant difference appeared between sexes in the total burnout score. Female athletes even reported higher levels of stress and a lower sense of personal accomplishment compared with male athletes. Sex differences in protective factors that frequently have been reported in previous studies is that of females scoring higher on ‘social resources’ and males scoring higher on ‘perception of self’ [34,36,41,50]. These sex differences may indicate that males and females tend to employ different strategies for coping in times of elevated stress [50]. Females may perceive healthy social environments as a greater resource than males, while males may depend on intrapersonal resources to a greater extent than females. In this context, both sexes may be equally vulnerable to the development of athlete burnout depending on the availability of these sex-specific protective factors. This interpretation fits well with the relative importance of sex (rank 12) in explaining athlete burnout.

The unique association between level of ambition and athlete burnout suggests that athletes with no ambitions of a future career in elite sport are more likely to experience burnout symptoms than athletes with ambitions to become future elite athletes. In line with this result, athletes with no elite ambitions reported lower-quality relationships with their coaches, lower levels of protective factors of resilience, higher levels of stress and more signs of athlete burnout compared with athletes with elite ambitions. Competitive sport represents an arena where individuals can learn to identify and overcome stressful situations [32] and, most likely, athletes with elite ambitions experience more stress and challenge related to their sporting activities than athletes with no elite ambitions. In the framework of exposure therapy, frightening (yet realistically safe) stimuli are presented repeatedly over time until the anxiety is reduced [51]. Likewise, exposure to stress—in a safe environment provided by the coach—may help young athletes to acquire the skills necessary for successful stress management. To the best of the authors’ knowledge, this is the first study to show that elite ambitions are uniquely associated with lower levels of burnout symptoms in young athletes.

### 4.2. The Importance of a Close, Personal Bond between the Coach and the Athlete

The bond dimension emerged as a unique, negative contributor to athlete burnout, indicating that the higher the level of bonding between the coach and the athlete, the lower the degree of athlete burnout. The bond dimension was also found to have the fourth strongest relative influence on athlete burnout, as shown by the dominance analysis. Additionally, the bond subscale exhibited strong inter-scale correlations with the goal (r_s_ = 0.74) and task (r_s_ = 0.81) subscales. Consistent with these results, athletes who reported higher-quality relationships with their coaches, reported higher levels of protective factors of resilience, lower levels of stress and less signs of athlete burnout. These findings are in line with previous work [16,52] and provide support for H2 in that a strong coach–athlete working alliance is associated with lower levels of burnout symptoms in young athletes. An empathic understanding of the athlete’s world makes it possible for the coach to communicate his/her understanding of the athlete’s experience [53]. An athlete must learn how to cope effectively with distress, and the coach may play an important role in this respect by helping the athlete to recognize, understand and change the specific patterns of thought and behaviour that evoke these subjective, negative stress reactions. 

The addition of the working alliance led to a significant improvement in explained variance (Model 2, ΔR^2^ = 0.19), suggesting that the nature of the coach–athlete working alliance is central when seeking to explain burnout in young athletes. The goal- and task dimensions, however, did not exhibit unique associations with athlete burnout in the full model, which could be explained by the strong inter-scale correlations between the three subscales of the WAI as well as the moderate correlations between the goal-and task subscales and (a) the RSA factor ‘planned future’ (r_s_ = 0.40, 0.43) and (b) perceived stress (r_s_ = −0.42, −0.41). Variance inflation factor (VIF) values did not indicate any multicollinearity problem. From another point of view, the regression results and the correlations between the investigated variables would also seem to suggest that a dysfunctional coach–athlete working alliance has a negative impact on athletes’ psychological resilience, levels of stress and vulnerability to burnout. The present study thus provides additional support for the idea that the coach–athlete relationship in fact is a ‘double-edged sword’—depending on the quality of this relationship, it may either contribute to decrease or increase an athlete’s level of stress and accordingly, prevent or promote the development of burnout [16].

### 4.3. The Importance of Psychological Resilience in Athletes

The protective factors ‘planned future’ and ‘structured style’ had unique, negative effects on athlete burnout, suggesting that the higher the levels of these protective factors, the lower the level of burnout symptoms. ‘Planned future’ was also found to have the second strongest relative influence on athlete burnout. Interestingly, the planning and organizing skills measured in these two subscales of the RSA seem to be closely associated with some aspects of the higher-level cognitive skills called executive functions (EFs) [54]. EFs can be described as the ability to manage and regulate one’s thoughts, emotions and behaviours in order to achieve desired goals, including elements, such as inhibitory control, working memory, cognitive flexibility, planning, problem solving, attention and reasoning [54]. EFs are important for aspects of learning and everyday performance, and previous work within the sporting domain has demonstrated that EFs are important in soccer and can even predict future success in soccer players [55]. EFs have further been suggested to provide a foundation for successful stress management [56]. Importantly, the prefrontal cortex (PFC) is a key structure of the brain underlying top-down ‘executive control’ [57]. The PFC is, however, among the most vulnerable brain regions to the adverse effects of chronic stress [58,59]. Evidence suggests that acute stress impairs the top-down regulation from the PFC, mechanisms that are further exaggerated by chronic stress [58,59]. A progressive loss of top-down cognitive control may also include impairments in the ability to cope with stress [58]. Considering the close association between EFs and the planning & organizing skills measured in ‘planned future’ and ‘structured style’, it can be hypothesized that chronic stress leads to a gradual loss of these protective skills, which further impedes adaptive coping with stress and accelerates the development of athlete burnout. In line with the current findings, ‘planned future’ and ‘structured style’ may, therefore, be essential factors in the explanation of athlete burnout. Overall, the present findings substantiate previous literature [26,28,60] and lend support to H2 in that higher levels of protective factors of resilience are associated with lower levels of burnout symptoms in young athletes.

The addition of psychological resilience further improved the explanatory power of the regression model (Model 3, ΔR^2^ = 0.13). Although present in Model 3, the unique effects of ‘perception of self’ and ‘social resources’ were no longer present in the full model. Possibly, these observations could be explained by the moderate to strong correlations between these protective factors and perceived stress (r_s_ = −0.65, −0.41), although VIF values did not indicate any multicollinearity. Interestingly, the RSA factor ‘perception of self’ has been found to be highly correlated with the Big Five personality factor ‘emotional stability’ (r = 0.79), suggesting that this protective factor is essential to counteract psychological vulnerability in the face of stress [40]. Supporting this idea, ‘perception of self’ exhibited the strongest correlation with perceived stress (r_s_ = −0.65) among all investigated variables. This interpretation also fits well with the relative importance of ‘perception of self’ (rank 3) in explaining athlete burnout. As for the unique association between ‘social resources’ and athlete burnout in model 3, various studies have identified social support to be a major determinant of athletes’ ability to cope effectively with stress [60,61].

### 4.4. The Importance of Reacting Positively to Stress

Perceived stress emerged as a unique, positive contributor to athlete burnout, indicating that the more stressful athletes perceive their life situations to be, the higher the degree of athlete burnout. Consistent with this result, athletes who reported higher levels of stress, reported more signs of athlete burnout. Among the investigated variables, perceived stress exhibited the strongest correlation with athlete burnout (r_s_ = 0.53) and was also found to have the strongest relative influence on athlete burnout. These results concur well with both previous findings [7,8,9,10] and Smith’s cognitive-affective model of athletic burnout [2], suggesting that stress is a major contributor to athlete burnout. These results further provide strong support for H2 in that higher levels of perceived stress are associated with higher levels of burnout symptoms in young athletes. 

The addition of perceived stress (Model 4) led to a relatively low, although significant, increase in R^2^ (ΔR^2^ = 0.04) compared with the addition of the coach–athlete working alliance and psychological resilience. This observation would appear to indicate a large proportion of shared variance between perceived stress and the other variables in Model 4, especially psychological resilience. 

The stressors encountered in sport are most often subjective rather than objective and universal, and accordingly, the nature of the stress response is mostly determined by the athlete’s personal interpretation [29]. It then follows that any factor that influences the cognitive appraisals of stressors is likely to affect the balance between eustress and distress and, thus, contribute to determine an athlete’s susceptibility to burnout.

### 4.5. Limitations

The strengths of the present study include its sufficiently large sample size, the equal representation of male and female athletes and the fairly wide selection of sports. Most importantly, however, the uniqueness of this study lies in the specific set of independent variables used to explain individual differences in athlete burnout. The relatively low response rate of 35% might be indicative of a nonresponse bias within the sample. However, the response rate alone is not necessarily a reliable measure of research quality [62]. It should also be noted that the relatively low response rate of 35% is consistent with the general, downward trend in respondent cooperation in cross-sectional surveys [63]. Further, this study may be limited by the features of a cross-sectional, correlational design. Although our interpretations are based on theory and previous work, cross-sectional, correlational research cannot support conclusions on causal relationships [64,65]. In addition, our data was self-reported and one does not know the extent to which self-reported data represents an accurate, unbiased reflection of what is being measured [66].

## 5. Conclusions

To build an effective and burnout preventive coach–athlete working alliance, a close, personal bond between the coach and the athlete seems to be fundamental. A coach who is genuine, accepting, trustworthy and empathic is better able to assess the subjective experience of the athlete and, therefore, better qualified to ensure an optimal balance between situational demands and intra- and interpersonal resources. An empathic coach is, therefore, more likely to succeed in building resilient athletes who are capable of realizing their potential in sport. An effective coach–athlete working alliance—characterized by personal bonding as well as coach–athlete agreement on goals and tasks—would not only prevent distress related to the relationship itself but also provide the athlete with adequate interpersonal resources, which may further facilitate the development of the athlete’s intrapersonal protective factors of resilience. Among these, planning and organizing skills such as the ability to formulate clear goals, organize and plan ahead, on a daily- and long-term basis, seem to be major determinants of the young athlete’s ability to cope with stress. Providing athletes with the tools and understanding necessary to interpret stressors in a positive way will make them more prone to react to stressors with eustress rather than distress.

## Figures and Tables

**Table 1:** Correlations, descriptive statistics, and Cronbach’s alphas of the variables, N = 670.

Variable	1	2	3	4	5	6	7	8	9	10	11	12	13
1. Level of ambition	–	–	–										
2. Sex	–	–	–										
3. WAI-S: Bond	–	–	–										
4. WAI-S: Goal	–	–	0.74	–									
5. WAI-S: Task	–	–	0.81	0.78	–								
6. RSA: Perception of self	–	–	0.33	0.29	0.36	–							
7. RSA: Planned future	–	–	0.40	0.40	0.43	0.61	–						
8. RSA: Social competence	–	–	0.19	0.14	0.22	0.38	0.28	–					
9. RSA: Family cohesion	–	–	0.26	0.29	0.27	0.39	0.39	0.33	–				
10. RSA: Social resources	–	–	0.34	0.32	0.35	0.47	0.45	0.42	0.66	–			
11. RSA: Structured style	–	–	0.21	0.26	0.26	0.35	0.42	0.22	0.33	0.38	–		
12. PSS: Perceived stress	–	–	−0.39	−0.42	−0.41	−0.65	−0.51	−0.25	−0.35	−0.41	−0.29	–	
13. ABQ: Global burnout	–	–	−0.46	−0.46	−0.48	−0.46	−0.49	−0.22	−0.31	−0.38	−0.32	0.53	–
Max. score	2	2	7	7	7	7	7	7	7	7	7	52	72
Min. score	1	1	1	1.50	1	1	1	1.33	1.33	2.14	1	4	15
M	1.22	1.51	5.50	5.28	5.18	4.91	5.20	5.12	5.76	6.13	5.12	24.46	36.11
SD	0.41	0.50	1.42	1.16	1.30	1.16	1.27	1.10	0.99	0.86	1.20	7.69	10.11
α	–	–	0.73	0.92	0.90	0.79	0.77	0.76	0.79	0.81	0.63	0.84	0.90
No. items	1	1	4	4	4	6	4	6	6	7	4	14	15

Note. *p* < 0.01; M = Mean; SD = Standard deviation; α = Cronbach’s alpha; Computations are based on cross-sectional data collected from 670 Norwegian junior athletes.

**Table 2:** Summary of independent-samples t-test results showing mean scores and standard deviations by level of ambition.

Variable	Elite Ambitions (N = 516)		No Elite Ambitions (N = 144)		*p*
	M	SD	M	SD	
WAI-S: Bond	5.72	1.26	4.69	1.65	***
WAI-S: Goal	5.44	1.10	4.69	1.18	***
WAI-S: Task	5.38	1.18	4.47	1.47	***
RSA: Perception of self	4.97	1.12	4.64	1.26	**
RSA: Planned future	5.36	1.19	4.68	1.39	***
RSA: Family cohesion	5.80	0.99	5.62	0.97	*
RSA: Social resources	6.17	0.84	6.00	0.92	*
RSA: Structured style	5.22	1.16	4.77	1.30	***
PSS: Perceived stress	23.81	7.40	26.87	8.32	***
ABQ: Emotional and physical exhaustion	11.39	4.03	13.02	3.85	***
ABQ: Reduced sense of accomplishment	12.84	4.01	15.26	3.69	***
ABQ: Sport devaluation	10.10	3.51	14.28	4.16	***
ABQ: Global burnout score	34.33	9.59	42.56	9.51	***

Note. *** *p* < 0.0005; ** *p* < 0.01; * *p* < 0.05.

**Table 3:** Summary of independent-samples t-test results showing mean scores and standard deviations by sex.

Variable	Male Athletes (N = 330)		Female Athletes (N = 340)		*p*
	M	SD	M	SD	
RSA: Perception of self	5.18	1.02	4.64	1.23	***
RSA: Planned future	5.36	1.21	5.04	1.30	**
RSA: Social resources	6.05	0.89	6.20	0.83	*
RSA: Structured style	4.94	1.21	5.31	1.16	***
PSS: Perceived stress	22.82	7.10	26.06	7.92	***
ABQ: Reduced sense of accomplishment	12.87	3.79	13.84	4.23	**

Note. *** *p* < 0.0005; ** *p* < 0.01; * *p* < 0.05.

**Table 4:** Summary of hierarchical multiple regression analysis results showing the unique and collective contributions of the coach- athlete working alliance, psychological resilience and perceived stress to the explanation of athlete burnout over and above level of ambition and sex alone.

Variable	Model 1			Model 2			Model 3			Model 4		
	B	SE B	β	B	SE B	β	B	SE B	β	B	SE B	β
Level of ambition	8.22	0.91	0.34***	4.81	0.85	0.20***	4.42	0.78	0.18***	4.41	0.75	0.18***
Sex	0.03	0.75	0.001	0.06	0.67	0.003	−0.82	0.66	−0.04	−1.41	0.65	−0.07*
WAI-S: Bond				−1.09	0.45	−0.15*	−1.00	0.41	−0.14*	−0.87	0.40	−0.12*
WAI-S: Goal				−1.32	0.49	−0.15**	−1.02	0.45	−0.12*	−0.56	0.44	−0.06
WAI-S: Task				−1.42	0.52	−0.18**	−0.29	0.48	−0.04	−0.36	0.46	−0.05
RSA: Perception of self							−1.92	0.37	−0.22***	−0.66	0.40	−0.08
RSA: Planned future							−1.14	0.34	−0.14**	−0.87	0.33	−0.11**
RSA: Social comp.							0.03	0.32	0.003	−0.07	0.31	−0.007
RSA: Family cohesion							0.16	0.42	0.02	0.18	0.40	0.02
RSA: Social resources							−1.06	0.52	−0.09*	−0.73	0.50	−0.06
RSA: Structured style							−0.68	0.30	−0.08*	−0.57	0.29	−0.07*
PSS: Perceived stress										0.39	0.06	0.30***
R^2^	0.11			0.30			0.43			0.47		
ΔR^2^	–			0.19			0.13			0.04		
Adj. R^2^	0.11			0.29			0.42			0.46		

Note. *** *p* < 0.0005; ** *p* < 0.01; * *p* < 0.05.

**Table 5:** Summary of dominance analysis results showing general dominance weights, general dominance percentages of model R^2^ and ranking of variables in terms of their relative influence on athlete burnout.

Variable	GDW	GD % of R^2^	Rank
Level of ambition	0.045	9.6%	6
Sex	0.003	0.6%	12
WAI-S: Bond	0.049	10.4%	4
WAI-S: Goal	0.043	9.2%	7
WAI-S: Task	0.047	9.9%	5
RSA: Perception of self	0.049	10.6%	3
RSA: Planned future	0.055	11.6%	2
RSA: Social competence	0.010	2%	11
RSA: Family cohesion	0.014	3%	10
RSA: Social resources	0.026	5.6%	9
RSA: Structured style	0.027	5.7%	8
PSS: Perceived stress	0.102	21.8%	1
Total model R^2^	0.47	100%	–

Note. GDW = General dominance weight, which represents the average additional contribution of an independent variable to R^2^ across all possible subset models; GD% of R^2^ = General dominance percentage of R^2^, which represents the percentage of model R^2^ associated with each independent variable.
